# Selfing and Drought-Stress Strategies Under Water Deficit for Two Herbaceous Species in the South American Andes

**DOI:** 10.3389/fpls.2019.01595

**Published:** 2019-12-17

**Authors:** Natalia Ricote, Cristina C. Bastias, Fernando Valladares, Fernanda Pérez, Francisco Bozinovic

**Affiliations:** ^1^ Departmento Ecología, Facultad de Ciencias Biológicas, Pontificia Universidad Católica de Chile, Santiago, Chile; ^2^ Center of Applied Ecology & Sustainability (CAPES), Pontificia Universidad Católica de Chile, Santiago, Chile; ^3^ Departamento de Biogeografía y Cambio Global, Museo Nacional de Ciencias Naturales (MNCN)–CSIC, Madrid, Spain; ^4^ Área de Biodiversidad y Conservación, Universidad Rey Juan Carlos, Móstoles, Spain

**Keywords:** selfing, drought, mating system evolution, global change, *Schizanthus*

## Abstract

Angiosperms are highly diverse in their reproductive systems, including predominantly selfing, exclusive outcrossing, and mixed mating systems. Even though selfing can have negative consequences on natural populations, it has been proposed that plants having a predominantly selfing strategy are also associated with fast development strategies through time limitation mechanisms that allow them to complete their life cycle before the onset of severe drought. This relationship might be affected by the challenges imposed by global change, such as a decrease in pollinator availability and the earlier and more severe onset of droughts. In this work, our aim was to investigate whether selfing is correlated with a dehydration avoidance strategy, and how this could affect drought resistance and survival in two species with different types of selfing: pollinator-independent delayed selfing (*Schizanthus grahamii*) and pollinator-dependent selfing (*Schizanthus hookeri*), representing a gradient in selfing rates. We hypothesize that delayed selfing species and highly selfing populations will show “fast” plant traits whereas we will find no pattern in more outcrossed populations of the pollinator-dependent species. However, we predicted that high selfing populations would have lower survival rates when exposed to chronic drought early in their development since fast traits imply physiological compromises that will affect their drought survival. To evaluate these hypotheses, we characterized different physiological and morphological traits in response to two contrasting treatments (moist and dry) in a total of six populations of the two species. We found a relationship between the delayed selfing species and a dehydration avoidance strategy and also with low drought survival. Our work offers evidence to support the importance of abiotic factors, such as drought, on the possible variation in selfing rates on natural populations, and the effect that this mating system could have in their ability to face new environmental conditions such as those imposed by climate change.

## Introduction

Mating system variation is common within and among angiosperms (Stebbins, 1974; Barrett, 1990), including not only alternative extremes of predominant outcrossing, or selfing, but also "mixed mating" systems present in ~40% of flowering plants (Goodwillie et al., 2005; Dudley et al., 2012; Moeller et al., 2017).

Within the 60% of potentially selfing species (Goodwillie et al., 2005; Jordan and Otto, 2012), there is pollinator independent (autonomous) selfing, such as delayed selfing, where the anthers touch the stigma at the end of the season assuring reproduction when crossed pollination is not possible. On the other hand, there is pollinator dependent selfing (geitonogamy), where selfers are not able to self-pollinate without the intervention of a pollinator that can visit the same flower several times, or several flowers from the same plant (Pérez et al., 2009).

A wide variety of studies have described the advantages and disadvantages associated with selfing. On one hand, selfing has the advantage of reproductive assurance; (Lloyd, 1992; Fausto et al., 2001; Snell and Aarssen, 2005; Dudley et al., 2012; Pérez et al., 2013), and automatic selection (Fisher, 1941; Schoen et al., 1996; Harder and Wilson, 1998; Aarssen, 2000), accordingly, it has been mainly observed on colonizing plants such as: weeds, herbaceous, and annual species (Lloyd, 1980; Barrett et al., 1996). On the other hand, it leads to lower adaptive potential, increase the probability of inbreeding depression (Herlihy and Eckert, 2002), and lower genetic variability in natural populations, reducing their evolutionary potential and increasing extinction risks (Herlihy and Eckert, 2002; Moeller and Geber, 2005; Dierks et al., 2012; Dudley et al., 2012). These outcomes have led biologists to ask whether selfing is an evolutionary dead end (Dobzhansky, 1950; Stebbins, 1957; Stebbins, 1974; Schemske and Lande, 1985; Igic and Busch, 2013). The importance of these advantages and disadvantages in the maintenance of natural populations might be altered by the new challenges imposed by global change, which can modify several aspects on their environment.

One of the greatest challenges in the study of the effect of global change on plant populations is the non-additive interaction between several human-driven threats that have traditionally been studied separately (Vitousek, 1997; Matesanz et al., 2009; Reed et al., 2012). Among these threats, there are two important problems that affect animal-pollinated plant populations. First, climate change is causing and will continue to cause changes in rainfall patterns across the world such as a global decrease of precipitations and desertification. These changes are predicted for both mountain and valley areas and a consequent increase of seasonal droughts in these environments is expected (IPCC, 2019). On the other hand, several human-driven changes, such as biological invasions, fragmentation, and habitat loss will cause a decrease in pollinator populations as well as a decoupling of distribution ranges and phenology between pollinators and plants, all of which cause a decrease in pollinator availability on plant habitats (Eckert et al., 2010; Leimu et al., 2010). The combination of these two threats implies that, in many self-compatible species, selfing frequency will increase (Stebbins, 1957; Hauser and Loeschcke, 1996; Levin, 2010; Reed et al., 2012), and the consequences of this change may not only alter mating systems but also other floral and/or plant traits (Jordan and Otto, 2012) that could alter the way populations may face new climatic extremes such as increasing aridity. In this context, to assess the interplay between an increase in selfing and an increase in drought conditions for plant populations, might allow better understanding on how animal pollinated plants will respond to future environmental changes.

Historically, adaptations that allow plants to face drought have been divided into three main physiological strategies: drought escape, dehydration avoidance, and drought tolerance (Ludlow, 1989; Kooyers, 2015). However, this terminology has been recently revised by (Volaire, 2018) and it was suggested that it would be better described as dehydration escape, dehydration avoidance, and dehydration tolerance. Each of these strategies also implies compromises that allow plants to have better drought resistance or drought survival. Previous work has shown that selfing can be part of a dehydration escape strategy (Mazer et al., 2010; Dudley et al., 2012; Dudley et al., 2015) in the sense that selfing is associated with shorter life cycles. Within that strategy, there is also an expression of traits associated with dehydration avoidance, all of them have been defined as "fast" traits which allow plants to avoid the periods of highest drought and dehydration risk. Since this strategy is based on the rapid acquisition of resources in order to maximize water uptake and/or minimize water loss; it implies compromises that would lower plant drought survival if exposed to severe long-lasting drought (Volaire, 2018). It has been proposed that autogamous selfing has been favored in species with a dehydration escape strategy since both result in a faster life cycle, and that both could have evolved together (Dudley et al., 2015; Ivey et al., 2016; Emms et al., 2018), this association is also known as the "time limitation hypothesis" (Aarssen, 2000; Snell and Aarssen, 2005; Emms et al., 2018). Accordingly, comparative studies in various herbaceous genera (Aarssen, 2000; Snell and Aarssen, 2005; Dudley et al., 2012) have documented that selfing species tend to have faster growth rates, higher photosynthetic rates, higher stomatal conductance and lower water use efficiency than their outcrossing congeners with a dehydration avoidance strategy that, on the other hand, allows them a moderate drought resistance (Heschel and Riginos, 2005; Mazer et al., 2010; Wu et al., 2010; Volaire, 2018).

However, it is interesting to pay attention to species that have different forms of selfing since, in the case of autonomous selfing, this is considered an evolved strategy (Pérez et al., 2009), whereas variations in selfing due to geitonogamy would not reflect an evolution associated with a drought escape strategy as much as an immediate pollinator limitation. Species with mixed mating systems where selfing rates can vary between populations could also help us understand these relationships.

*Schizanthus hookeri* Gillies ex Graham and *Schizanthus grahamii* Gillies ex Hook (Solanaceae) are two annual and biannual herbs that grow in the Andes of Central Chile. Climate change scenarios for this region predict an increase of 4 to 5°C in temperatures and >40% of decrease in rainfall during growing season by the end of the present century (Fuenzalida et al., 2006). An increase in precipitation variability, and thus in the occurrence of extreme seasonal droughts, is also expected (Fuenzalida et al., 2006; IPCC, 2019).

Annual plants, such as *Schizanthus* species, are thought to be particularly vulnerable to these events since they have to complete their life cycle within a single season, and population persistence is highly dependent on the reproductive output of a season (Heschel et al., 2004). Both *Schizanthus* species are self-compatible, but only *S. grahamii* has the ability for autonomous selfing [i.e., without vector intervention; (Pérez et al., 2009)]. Selfing in *S. grahamii* occurs after the opportunity for outcrossing has passed (delayed selfing), and the rate varies strongly with pollinator availability (Pérez et al., 2013); in this species, selfing also shows floral traits that are both associated with drought escape and selfing, such as smaller flowers that mature faster and allow for easier self-fertilization (Elle, 2004; Pérez et al., 2009; Pérez, 2011). *S. hookeri* is also self-compatible, but shows herkogamy and dichogamy, and thus requires pollinators for seed set. Selfing in this species can only occur when pollinators transfer pollen among flowers of the same plant (geitonogamy), therefore in this species, selfing is not associated with any specific floral traits. Selfing rates vary strongly between populations of these *Schizanthus* species with values ranging from 0.23 to 0.85 and are also associated with floral morphology, especially for the population with the highest selfing rate. These rates are likely to increase with a reduction in population sizes and mate availability (Pérez et al., 2013).

In this study, we asked whether selfing rate is related to dehydration avoidance, and, given the trade-offs of different physiological strategies, whether a higher selfing rate would negatively affect the capacity of highly selfing populations to display dehydration tolerance traits, facing a possible future scenario where the onset of drought could be earlier in their development. For that, we aimed to investigate 1) whether there is a linear relationship between selfing rate and key traits related to dehydration avoidance, and 2) whether there is a difference between species with different types of selfing regarding this physiological strategy. We studied six populations of *S. hookeri* and *S. grahamii* with contrasting selfing rates and conducted a greenhouse experiment to compare these species and assess how populations respond to drought conditions. We also measured morphological traits related with physiological responses to drought.

For aim one, we predicted that populations with high selfing would show dehydration avoidance traits, such as high photosynthetic rate and growth rate, but that, when exposed to drought conditions, would show decreased physiological responses to tolerate drought stress, such as low water use efficiency and high specific leaf area. We also predicted lower survival rates in high-selfing populations than outcrossing counterparts when exposed to chronic drought throughout the entire life cycle. For aim two, we hypothesized that only species that have evolved a delayed selfing strategy would display dehydration avoidance, since there is no mechanism associated with the time limitation hypothesis by which dehydration escape might lead to increased selfing in the species with geitonogamy.

## Materials and Methods

### Study Species

*S. hookeri* Gillies ex Graham and *S. grahamii* Gillies ex Hook are two annual, and occasionally biennial, herbaceous sister species of the *Schizanthus* genus. These two species are endemic to Chile and Argentina (Grau and Gronbach, 1984) and currently they share a similar distribution range across the Chilean Andes: *S. grahamii* grows at high elevations between 33 and 39°S and *S. hookeri* grows at mid- and high elevations between 29 and 38°S (Grau and Gronbach, 1984). The life cycle of these two herbaceous species lasts approximately 3 months during the austral summer, corresponding to the hot and dry season of the year in this ecosystem. Each species represents a reproductive system that can generate selfing in different ways: *S. grahamii* is mainly pollinated by hummingbirds and exhibits delayed selfing. This means its style elongates throughout flower development, and once the opportunity for outcrossing has passed, the stigma makes contact with the anthers, allowing selfing to occur and making it possible for selfing to happen with and without the presence of pollinators. On the other hand, *S. hookeri* is also self-compatible, but presents strong herkogamy and dichogamy and therefore requires pollinators for seed set and selfing. Its main pollinators are bees, dipterans and also hummingbirds (Pérez et al., 2013). Seeds are produced in both species inside several capsules per plant and usually fall down on the ground near mother plant without apparent signs of dispersion by wind or animals (Pérez et al., 2013).

### Seed Collection

We collected a sample of 50 seeds from 50 random individuals belonging to three populations of *S. grahamii* and three populations of *S. hookeri*. Populations were located between 33 and 34°S in the high Andes of Central Chile at elevations ranging between 2,000 and 2,500 m and within a maximum linear distance of 200 km (**Table 1**). Climatic conditions of these sites are similar, with mean annual precipitation of 900 mm, falling predominantly as snow between May and September, and a mean growing season temperature ranging between 9 and 15°C [(Cavieres et al., 2000), Centro de Clima y Resiliencia; see **Table S1**]. These sites included one location where populations of the two species occur sympatrically (Laguna los Cristales). Selfing rates for these populations were previously estimated by (Pérez et al., 2009), using microsatellite markers and a multilocus approach based on heterozygosity disequilibrium values (**Table 1**). Methods based on the degree of heterozygosity provide a long-term measure of the degree of selfing, reflecting a historical average as opposed to progeny-array methods, which base the estimation of selfing rates on the genetic analysis of progeny for which one or more parents are known, therefore focusing on short-term measures (Milligan and Strand, 1996; Ritland, 2002). Additionally, previous studies have described an association between populations with high selfing rates and floral morphology in the delayed selfing species that suggest that variation in selfing among populations in this particular species represents an evolved difference.

**Table 1:** Selfing rates at six microsatellite loci for three populations of the delayed autonomous selfing *Schizanthus grahamii* and three populations of non-autonomous self-compatible congeneric *Schizanthus hookeri.* Data from Pérez et al. (2013). For each population, location data (latitude, longitude, and elevation) and selfing rate (s(Fis); estimated from F_is_ = inbreeding coefficient) are shown.

Populations	Location (lat, lon, elevation)	s(Fis)
***S. hookeri***		
**Lagunillas (LA)**	33°36' S, 70°17'W, 2250 m	0.29
**Laguna de los Cristales (LC-H)** **Valle Nevado (VN)**	34°34'S, 70°32'W, 2360 m 33°21'S, 70°17'W, 2450 m	0.38 0.23
***S. grahamii***		
**La Parva (PA)**	33°19'S, 70°17'W, 2350 m	0.85
**Laguna los Cristales -G (LC-G)**	34°34'S, 70°32'W, 2360 m	0.43
**Teno (TE)**	35°11'S, 70°30'W, 2170 m	0.54

### Greenhouse Experimental Design

#### Seed Sowing

In April 2014, 45 seeds from 20 random mothers per population were stratified for 24 h in water and then germinated in sterilized Petri dishes containing filter paper soaked with a solution of gibberellin at a concentration of 100 ppm in order to stimulate growth. Petri dishes were sealed with Parafilm to prevent evaporation of water, thus avoiding changes in concentration of the solution. Seeds were incubated in growth chambers at 7°C for the first 4 days and at 20°C from the fourth day onwards. After 7 days, the seeds started to germinate (seeds were considered germinated with the emergence of the root). Finally, 11 germinated seeds from 15 mothers (which showed the best germination rates) per population were planted in plastic pots within trays of 15 pots each. Pots were filled with a 2:1:1 mixture of sterilized peat moss (Turba white 6F, Projar, Spain), vermiculite (type 3, Projar, Spain), and washed coarse sand (Leroy Merlin, Spain) and placed in a greenhouse under controlled conditions. The final study sample included *n =* 990 plants (6 populations x 15 mothers/population x 2 treatments x 11 replicates/mother). Life cycle of these species are approximately three to 4 months; therefore, to minimize confounding maternal effects, all seedlings were grown in the greenhouse under standard conditions of high water and nutrient availability for 1 month before starting the treatments (Lázaro-Nogal et al., 2015).

#### Treatments

Treatments were applied from May to August 2014, plants had a mean height of 8.11 cm (SD = 3.32 cm) when treatments started. Five replicate seedlings per mother were assigned to the control treatment and six replicate seedlings per mother were assigned to the drought treatment. Each replicate was assigned randomly to one of the six greenhouse benches to minimize micro environmental biases. Treatments simulated two contrasting regimes of water availability: moisture (control treatment) and drought, aimed to assess a range of conditions to which plants could be exposed in the future. In the moist treatment, plants were kept at field capacity, which, for our specific soil mixture was equivalent to 27–29% of soil water content (SWC). SWC was calculated as (W-D) x D-1, where W is the weight of the original sample and D is the weight of the dried sample. SWC was determined for a random subsample of 19 trays 16 times during the 2 months of experiment when the majority of measurements were made (June and July).

Throughout the duration of the experiment, all conditions in the greenhouse were monitored every 10 min with a HOBO H08-006-04 data logger (Onset, Pocasset, MA, USA) and plants were provided with all the nutrients needed. Mean temperature and minimum and maximum daily range for this period were: 21.2, 12–15, and 27–30°C, respectively. Photosynthetic active radiation (PAR) was between 800 and 1,000 μmol m^‑2^ s^‑1^.

### Physiological and Morphological Measurements

#### Performance Traits

We measured two traits (survival and daily growth rate) as proxies of plant performance. A survival curve was drawn for each population for both treatments, based on four mortality censuses made during the experiment, these censuses were performed at the same time as the other measurements, being the last census, at the time when the experiment ended (4 months after germination). Survival measure was the percentage of living individuals by the time the census was made. Plant height (elongation from the ground to the most recent node observable) of all plants was measured once a month. Growth was calculated as the growth rate per day between the measurements where there were greater differences (measurement 1 and 2), meaning, the moment of the highest growth rate, and was calculated as the rate: centimeter per day in 21 days.

#### Physiological and Water Economy Traits

Photosynthetic rate (μmol CO_2_ m^‑2^ s^‑1^), stomatal conductance to water vapor μmol H_2_O m^‑2^ s^‑1^, and iWUE (ratio between photosynthetic rate and stomatal conductance; μmol CO_2_ mol^‑1^ H_2_O^‑1^) are gas exchange traits affecting overall plant development rate and drought-tolerance (Chaves et al., 2003). These were measured on one fully-expanded leaf, randomly-selected of a primary branch per plant, using a Licor 6400 infrared gas analyzer (LI-COR, Lincoln, NE, USA); if the leaf measured was too small to fill the entire sensor, a correction was made to match the real occupied area. Leaves were exposed to a CO_2_ concentration of 400 μmol mol^‑1^ and saturating light of 1,500 μmol m^‑2^ s^‑1^ and measurements were made at temperatures between 24 and 26°C and relative humidity of approximately 30–50%. Measurements were made on a subsample consisting of four individuals per treatment belonging to five mothers per population.

#### Morphological Leaf Traits

Upon completion of the experiment, three fully expanded and mature leaves, randomly selected from a primary branch on each plant alive at the moment, were collected, and kept at 4°C, completely hydrated for 24 h. They were then weighed and scanned using a LI-3000C portable area meter (LI-COR). Finally, leaves were oven dried at 50°C for 48 h and weighed to determine specific leaf area (SLA), which is the ratio between leaf area and leaf dry biomass. Leaf water content was also calculated as: fresh weight-dry weight/dry weight.

### Statistical Analysis

To assess the differences in survival between populations and treatments, we used a log-rank test (also known as Mantel-Cox test). This test compares the slopes of survival curves computed from all the data in the curve, which was obtained from the mortality censuses described above. The explanatory variables considered included population (associated with a specific selfing rate) and treatment. For each curve a chi-square value was computed comparing the observed and expected number of deaths, the sum of all chi-square values gets an overall chi-square, from which P value is determined (Machin et al., 2006). Curves were analyzed and drawn with the program "GraphPad Prism version 5 for Windows, GraphPad Software, La Jolla California USA, www.graphpad.com."

For the other traits studied, including growth rate, SLA, leaf water content, and gas exchange traits (photosynthesis, stomatal conductance and water use efficiency), we performed ANOVA analysis on linear mixed models in order to test the effect of the explanatory variables. The random part of these models accounted for pseudo replication, since the experiments use multiple offspring from mother plants, which are not independent, and it also uses several mothers for each population. Therefore, the random structure of the models included mother, nested in population, nested in species. We performed model selection using Akaike information criterion (AIC) (Akaike, 1974; Akaike, 1998) selecting the best random structure that fit the data and then selecting relevant variables for the model to find the best-fit model both for the random and fixed structure. While the random structure was the same for all traits, the fixed structure was different depending on the trait and which explanatory variables rendered the most parsimonious model according to AIC criterion (Burnham and Anderson, 2002; Zuur et al., 2009). Analyses were performed using “nlme” and “lme4” libraries of the R Statistical Package (Pinheiro, 2009; Bates et al., 2014) using R version 3.5.1.

## Results

### Survival Analysis

**Figure 1:**
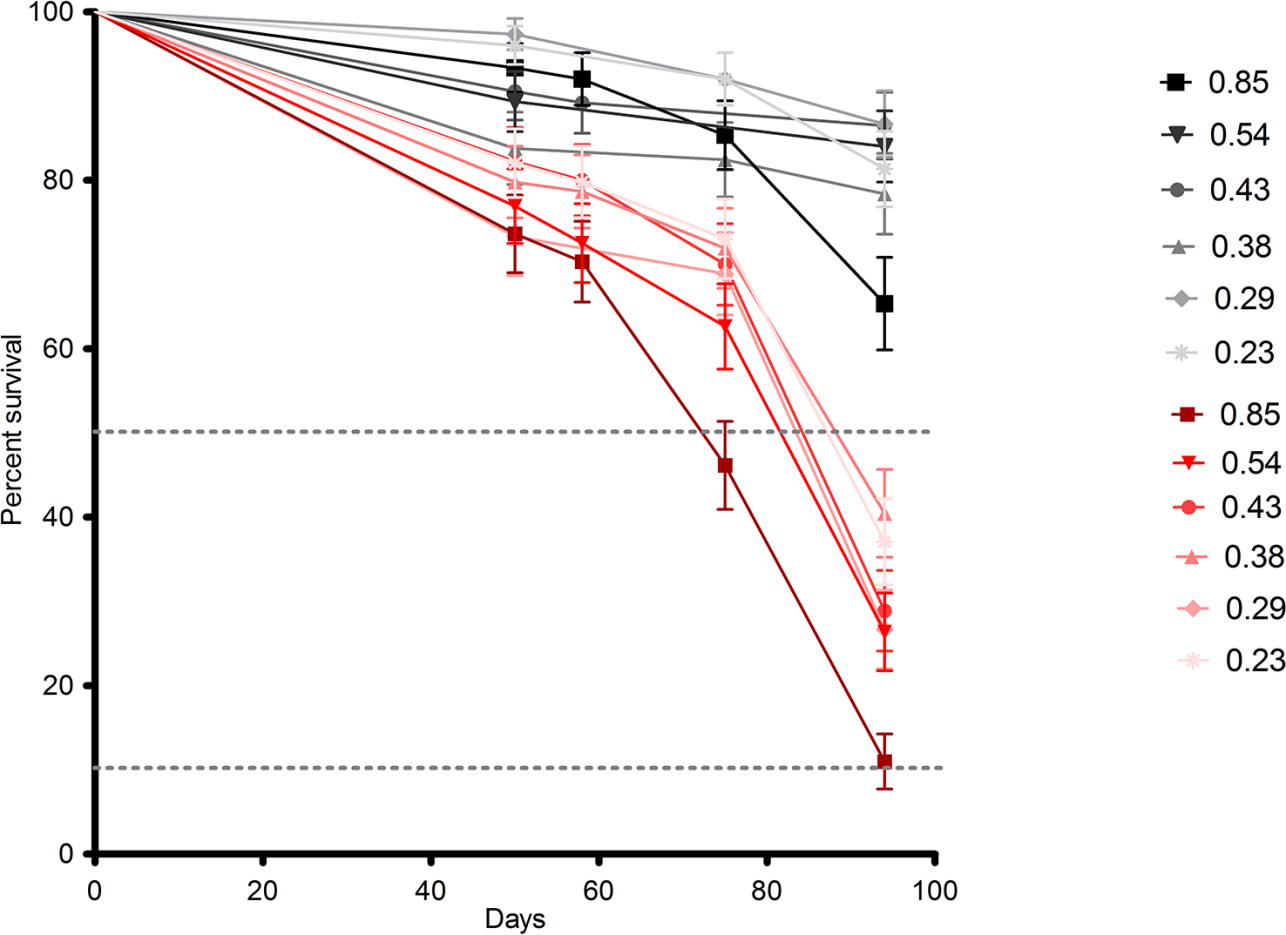
Survival curves representing the survival percentage for each population of the *Schizanthus* species: *S. hookeri* and *S. grahamii*, on the “wet” (black lines) and “dry” (red lines) treatments throughout mortality censuses performed during the experiment. Each population represents a specific selfing rate as indicated in the legend (see **Table 1**). The dotted lines represent 50 and 10% survival rate thresholds.

Our survival results show that both treatment and selfing are related with survival. Survival rates were significantly lower for all populations under drought treatment (*P =* 0.0001; **Figure 1**, in red), showing a negative effect of drought for the survival of all populations. On the other hand, the population with the highest selfing rate ("La Parva"; selfing rate = 0.85) had a significantly higher mortality rate at the end of the study (*p* < 0.001), with a survival percentage of only 11% in the dry treatment and of 65% in the wet treatment (**Table 2**). This population belongs to the delayed selfing species *S. grahamii*.

**Table 2:** Survival rates (%) of populations of *Schizanthus* species in the wet and dry treatments. Selfing rates for each population are indicated in parenthesis.

Population	Wet	Dry
***S. hookeri*** **LC-H (0.38)**	78.37%	40.44%
**VN (0.23)**	81.3%	37.07%
**Lag (0.29)**	86.66%	26.66%
***S. grahamii***		
**LC-G (0.43)**	86.48%	28.89%
**Te (0.54)**	84%	26.37%
**LP (0.85)**	65.3%	10.98%

LC-H., Laguna los Cristales, S. hookeri; VN, Valle Nevado; Lag, Lagunillas; LC-G, Laguna los Cristales S. grahamii; Te, Teno; LP, La Parva.

### Growth Rate Analysis

Best-fit model for growth rate included treatment, selfing and species as factors, and also the interaction between selfing and species. Selfing did not have a significant effect on growth rate (**Table 3**). However, species did have a significant effect on growth rate (F = 10.7, *p =* 0.002; **Table S2**). Because species was a nominal variable, one did not have an estimated coefficient (here *S. grahamii*) and was used only as a contrast for the other (*S. hookeri*). As the *S. hookeri* ($\bar{\text{x}}$ (mean) = 0.35, sd (standard deviation) = 0.2) had negative estimated coefficients, the individuals belonging to this species had lower daily growth rate than individuals from *S. grahamii* ($\bar{\text{x}}$= 0.37, sd = 0.17) (**Table 3**).

**Table 3:** Summary of the best-fit growth rate model, which included: treatment, selfing, species and the interaction between selfing and species.

Variable	Parameter estimated	*t* value	P-value
**Intercept**	5.58e^‑1^	8.95	4.82e^‑14^***
**Wet treatment**	7.05e^‑4^	0.07	0.94
**Selfing**	–3.09e^‑1^	–3.15	0.002**
**S.H.**	–3.48e^‑1^	–3.27	0.002**
**Selfing: S.H.**	7.71e^‑1^	2.58	0.01*

S.H., S. hookeri. Estimated parameters with their associated p-values are shown for all variables in the model (Selfing, treatment, species, the interaction term between selfing and species). A mixed model with a nested random intercept for each mother was generated (see in Statistical Analysis section). Significance levels: P > 0.05 (ns, not significant); P < 0.05 = *; P< 0.01 = **; P < 0.001 = ***

**Figure 2:**
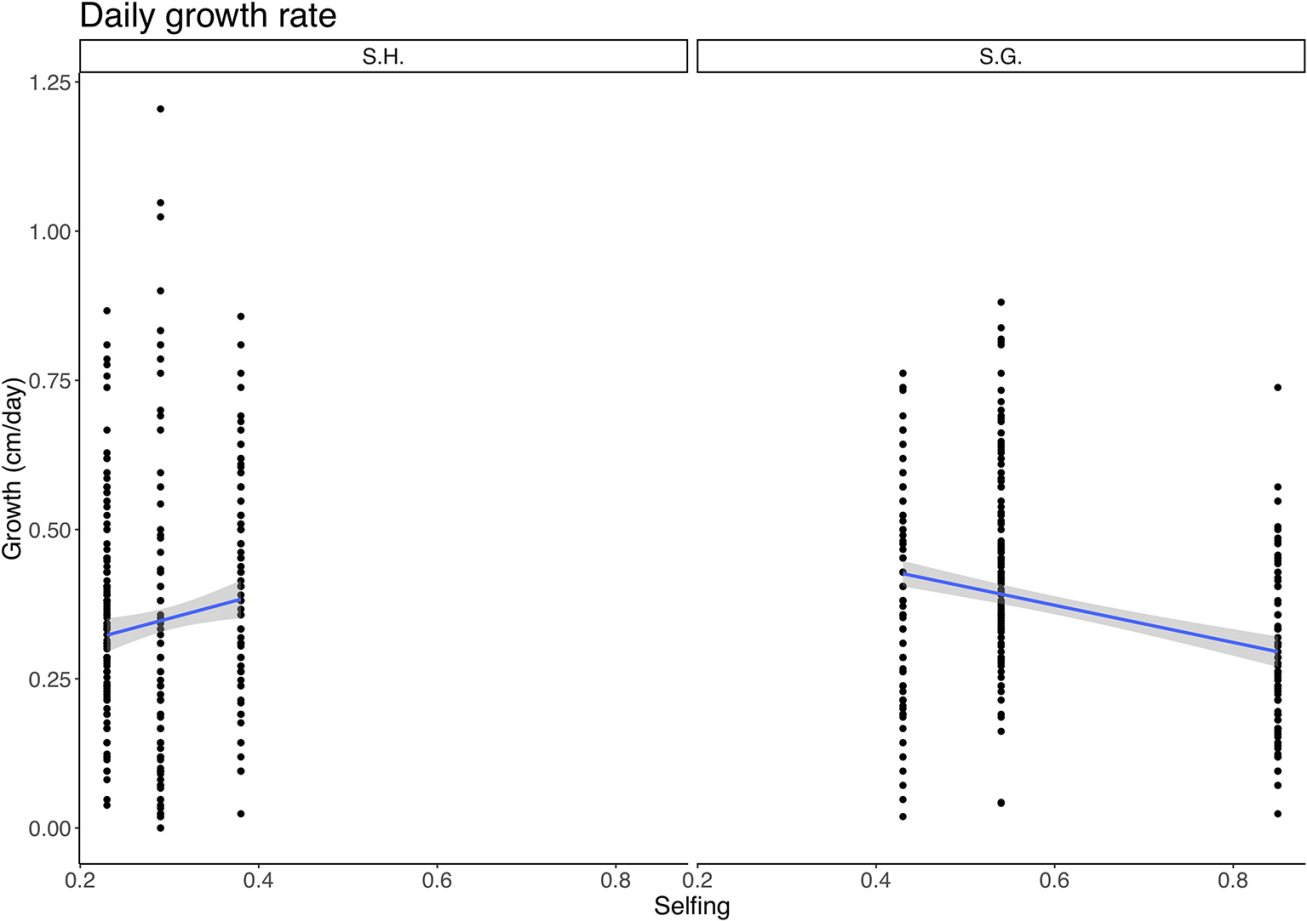
Relationship between daily growth rate (cm/day) and selfing rate for *Schizanthus hookeri* (left) and *Schizanthus grahamii* (right). Black dots are measurements for each selfing rate, blue lines represent linear relationships, and gray shaded area represents standard error. S.H., *Schizanthus hookeri*; S.G., *Schizanthus grahamii*.

Also, the interaction between species and selfing was significant (F = 6.66, p = 0.01) (**Table S2**). Within *S. grahamii*, higher selfing populations showed lower growth rates, whereas, in *S. hookeri* higher selfing populations had higher growth rates (**Figure 2**).

### Physiological and Water Economy Traits

**Figure 3:**
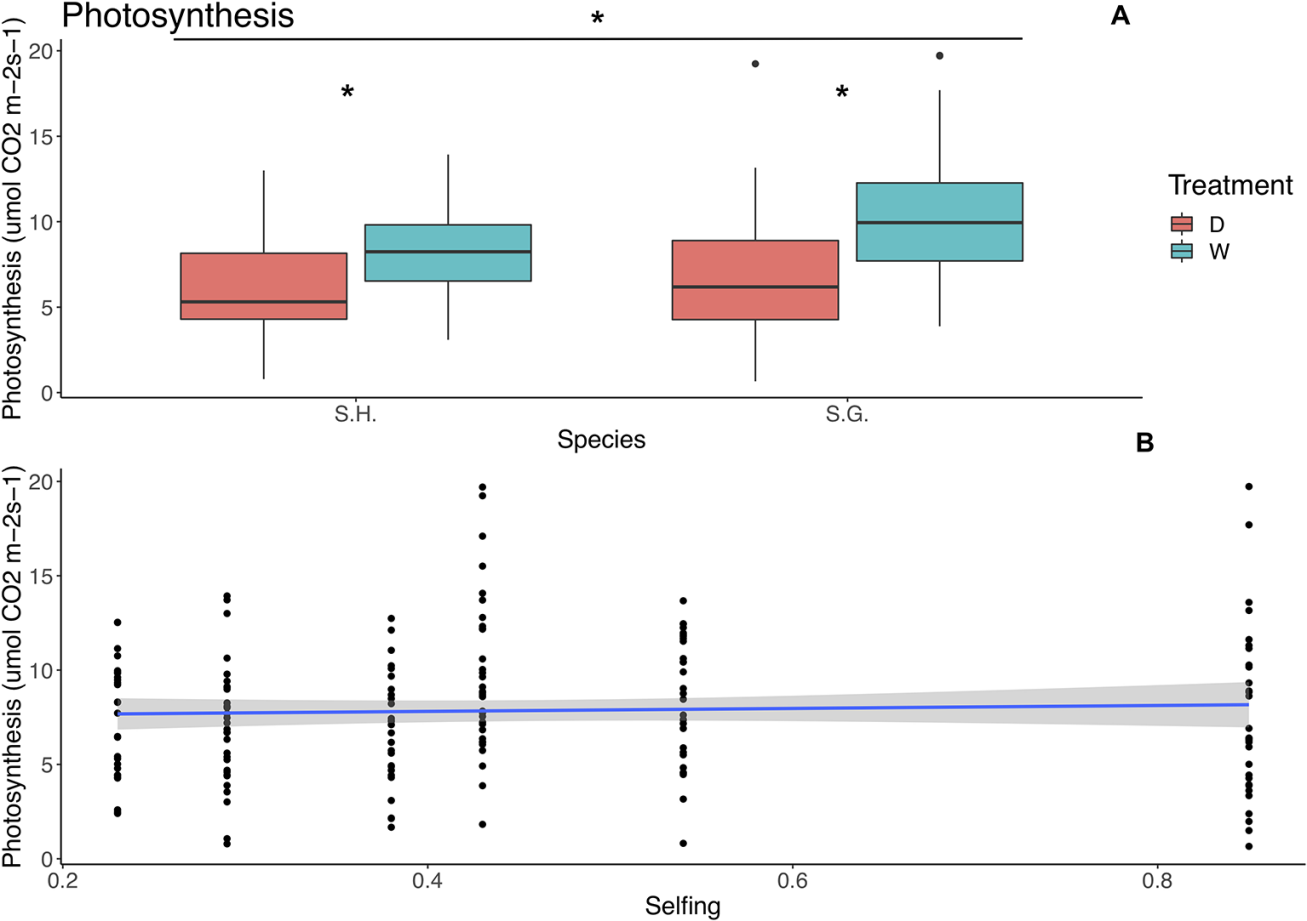
Relationship between photosynthetic rate (µmol CO^2^ m^‑2^ s^‑1^) and species (upper panel) and selfing rate (lower panel). **(A)** Red boxes represent the dry treatment and green boxes represent the wet treatment. S.H., *Schizanthus hookeri*; S.G., *Schizanthus grahamii*; D, dry treatment; W, wet treatment. **(B)** Blue line represents linear relationship and the gray area is the standard error. Significance level: *P < 0.01.

Selfing was included as a covariate in all of the best-fit models for photosynthesis, water use efficiency, and conductance; however, it was significant only for the photosynthesis model (F = 4.99, p = 0.04). For this trait, treatment and species were also considered as factors, but no interaction between these factors was included (**Table 4**). Treatment and species had a significant effect; photosynthesis was higher in *S. grahamii* ($\bar{\text{x}}$= 8.6, sd = 4.14) than in *S. hookeri* ($\bar{\text{x}}$= 7.07, sd = 3.09) and in the wet treatment (**Figure 3A**). Also, there is a significant negative linear relationship between selfing and photosynthesis (**Table 4**, **Figure 3B**).

**Table 4:** Summary of the best-fit photosynthesis model, which included: treatment, selfing, and species but no interactions between them.

Variable	Parameter estimated	*t* value	P
**Intercept**	9.86	7.38	7.26e^‑08^***
**Wet treatment**	2.96	6.14	6.37e^‑09^***
**Selfing**	–4.64	–2.23	0.035*
**S.H.**	–2.85	–3.37	0.003**

S.H., S. hookeri. Estimated parameters with their associated P values are shown for all variables in the model (selfing, treatment and species, no interaction was included). A mixed model with a nested random intercept for each mother was generated (see in Statistical Analysis section). Significance levels: P > 0.05 (ns, not significant); P < 0.05 = *; P< 0.01 = **; P < 0.001 = ***.

**Figure 4:**
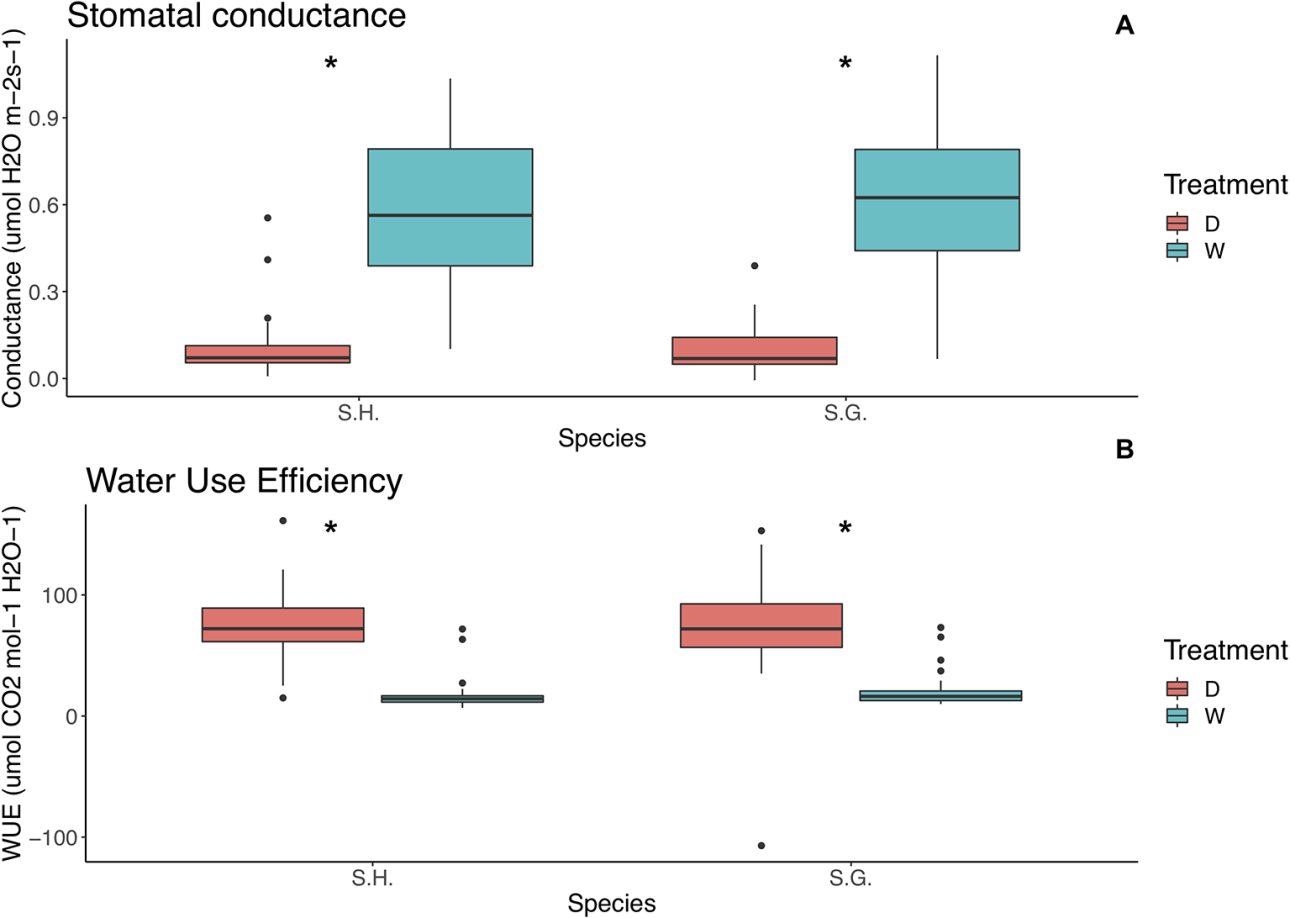
Relationship between **(A)** stomatal conductance (µmol H_2_O m^‑2^ s^‑1^) and species and **(B)** water use efficiency (µmol CO_2_ mol^‑1^H_2_O^‑1^) and species. Red boxes represent the dry treatment and green boxes represent the wet treatment. S.H., *Schizanthus hookeri*; S.G., *Schizanthus grahamii*; D, dry treatment; W, wet treatment.; WUE, water use efficiency. Significance level: *P < 0.01.

For both water use efficiency (WUE) and stomatal conductance, the only significant effect was treatment (wue: F = 221.4, p < 0.000001; conductance: F = 335.4, p < 0.000001), WUE was higher on the dry treatment ($\bar{\text{x}}$= 73.9, sd = 33.2) than on the wet treatment ($\bar{\text{x}}$= 18.5, sd = 12.4) regardless of species or selfing rate. On the other hand, conductance was higher on the wet treatment ($\bar{\text{x}}$ = 0.6, sd = 0.2) than on the dry ( $\bar{\text{x}}$ = 0.1 sd = 0.09), for all selfing rates and both species (**Figure 4**) (**Tables S3** and **S4**).

### Morphological Leaf Traits

**Figure 5:**
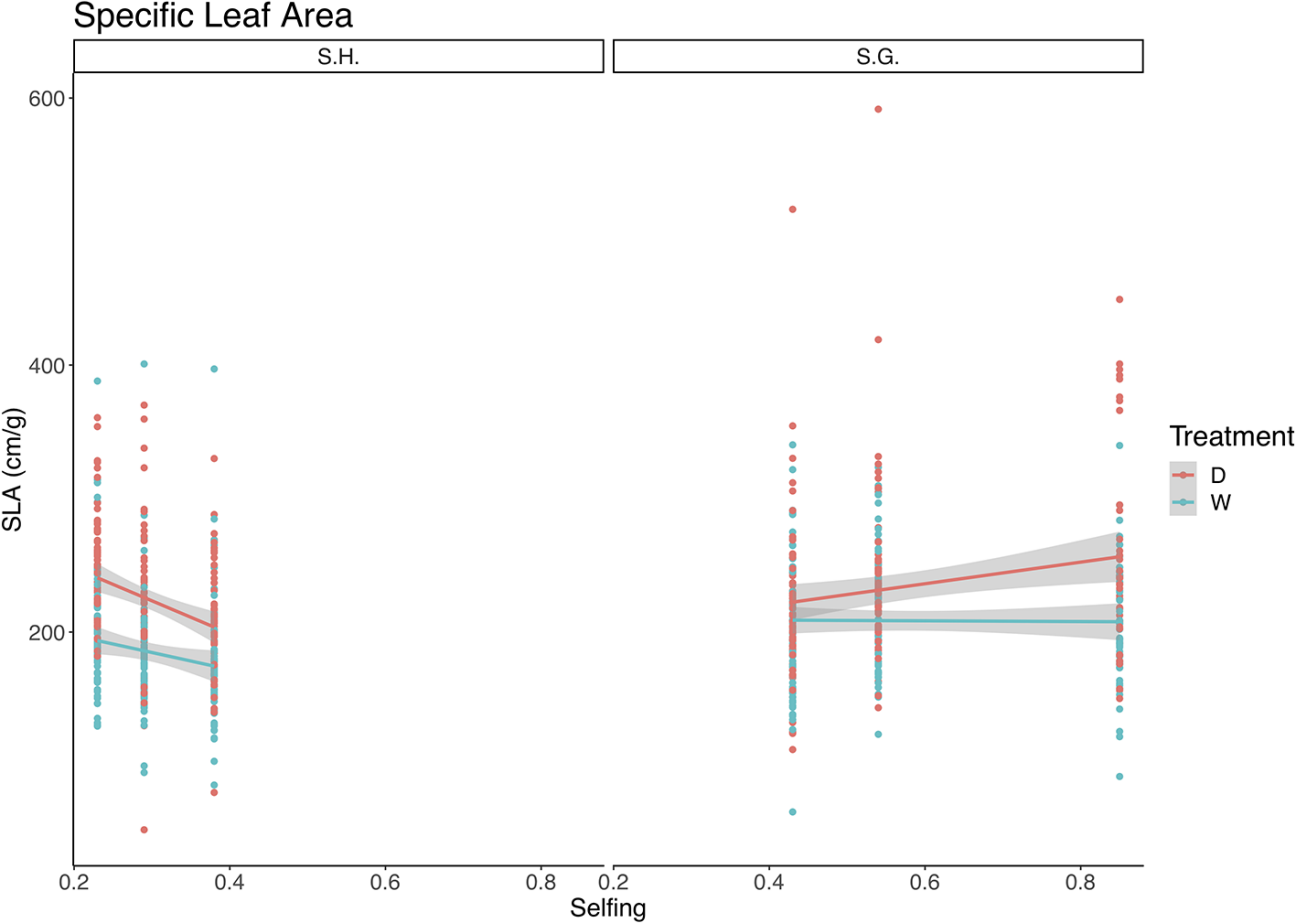
Relationship between specific leaf area (cm/g) and selfing rate for *Schizanthus hookeri* (left) and *Schizanthus grahamii* (right). Red dots are measurements for each selfing rate at the dry treatment and green dots are measurements for each selfing rate at the wet treatment, lines represent linear relationships (red: dry treatment, green: wet treatment), and gray shaded areas represent standard error. S.H., *Schizanthus hookeri*; S.G., *Schizanthus grahamii*; D, dry treatment; W, wet treatment.

The best-fit model for SLA included selfing as a covariate, treatment, and species as fixed factors and the interaction between all (**Table 5**). Both species and selfing had a significant effect on this trait, as well as the triple interaction of species, treatment, and selfing (**Table 5**). *S. grahamii* had higher SLA ($\bar{\text{x}}$= 221.5, sd = 60.5) than *S. hookeri* ($\bar{\text{x}}$= 203.9, sd = 52.7) and SLA was higher for the dry treatment than the wet. For *S. grahamii*, the difference between treatments was less pronounced than for *S. hookeri.* On the other hand, for *S. grahamii*, SLA increased with selfing rate, and this increase was more pronounced in the dry treatment, whereas for *S. hookeri*, SLA decreased with selfing for both treatments (**Figure 5**).

**Table 5:** Summary of the model that best fit specific leaf area data, which included: treatment, selfing, and species, and also all the interactions between them.

Variable	Parameter estimated	*t* value	P
**Intercept**	188.4	11.5	<2e^‑16^***
**Selfing**	79.6	2.98	0.003**
**Wet treatment**	22.43	1.14	0.25
**S.H.**	110.29	3.98	9.7e^‑05^***
**Selfing: wet treat**	–82.8	–2.56	0.01*
**Selfing: S.H.**	–329.6	–4.24	3.56e^‑05^***
**Wet Treat: S.H.**	–97.08	–2.95	0.003**
**Selfing: wet treat: S.H.**	204.8	2.2	0.03*

S.H., S. hookeri. Estimated parameters with their associated P values are shown for all variables in the model (selfing, treatment, and species, no interaction was included). A mixed model with a nested random intercept for each mother was generated (see is Statistical Analysis section). Significance levels: P > 0.05 (ns = not significant); P < 0.05 = *; P< 0.01 = **; P < 0.001 = ***.

The best-fit model for leaf water content included selfing as a covariate, treatment, and species as fixed factors and the interaction between selfing and species (**Table S5**). Both species and selfing had a significant effect on this trait, as well as the interaction of species and selfing (**Table S5**). *S. grahamii* had higher leaf water content ($\bar{\text{x}}$ =5.48, se (standard error) = 0.094] than *S. hookeri* ($\bar{\text{x}}$= 4.8, se = 0.17). On the other hand, for *S. grahamii*, leaf water content increased with selfing rate, whereas for *S. hookeri*, leaf water content decreased with selfing (**Figure S1**).

## Discussion

We found a somewhat consistent linear relationship between selfing and key traits associated with dehydration avoidance, providing some evidence to support the hypothesis that it is associated with a specific physiological profile. However, we also found that the differences appeared between species for most traits, indicating a stronger association of the delayed selfing species (*S. grahamii*) with a dehydration avoidance strategy than the geitonogamous species (*S. hookeri*). Some of our results suggest that this association implies a greater vulnerability to drought, since the compromises between strategies could mean high selfing species lack of physiological mechanisms to cope with drought conditions imposed earlier in their development, one of the results that suggests this is the lower drought survival observed for the populations with higher selfing rate (see below).

In the case of growth rate, the relationship between selfing and growth, depends on whether it is the delayed selfing species *S. grahamii* or the geitonogamous species *S. hookeri* since the interaction had a significant effect. The former showed a negative linear relationship between selfing and growth rate, meaning that the most selfing population within this species has the lowest growth rate, which does not support the "fast traits" hypothesis within the species. On the contrary, in the case of *S. hookeri*, selfing is positively related with growth rate. Therefore, we could only find signs of a dehydration avoidance strategy for the geitonogamous selfing species *S. hookeri* (**Figure 2**). Since treatment didn't have a significant effect on this trait, it would appear that growth rate in these populations is unlikely to be affected by immediate environmental conditions and it is probably more genetically determined. Nevertheless, we found a significant difference between species, with *S. grahamii* having an overall higher growth rate than *S. hookeri*, supporting the dehydration avoidance hypothesis. (*S. grahamii* not only has delayed selfing, it also has an overall higher selfing rate than *S. hookeri*). Growth-related traits are commonly used as proxies of fitness (Gianoli and González-Teuber, 2005; Lázaro-Nogal et al., 2015), therefore, a low growth rate for the population that shows the highest selfing rate, could be a sign of a general pattern of low performance due to inbreeding depression, although no specific test for it was performed.

Gas exchange rates in both species were most affected by lack of water. In the drought treatment plants were kept at 30% of field capacity, which is equivalent to 8.8% of SWC. Simulations of climate change scenarios in other studies of semi-arid ecosystems have reported reductions of SWC around 15% (Valladares et al., 2006).

We found a negative relationship between selfing and photosynthesis (**Table 4**), which was not consistent with a dehydration avoidance strategy that includes a faster life cycle, and therefore higher photosynthetic rates. However, the species with the overall highest selfing rate and a delayed selfing strategy (*S. grahamii*), had higher mean photosynthetic rates than the geitonogamous species, suggesting that the different strategies would be distinguished at the species level, between two species with two different evolutionary paths. Regarding treatment, photosynthesis was lower on the dry treatment for both species (**Table 4**, **Figure 3A**), therefore, if we are to expect earlier and drier summers in the future, it is possible that chronic drought such as the one simulated here, could occur in nature, which could lead to a decrease in photosynthetic rates in time and affect performance of these populations.

This wasn't the case for stomatal conductance to water vapor and water use efficiency; the drought treatment affected all populations in the same manner, mainly increasing their water use efficiency (**Figure 4B**). This particular trait has been known to be highly plastic and one of the fastest and more immediate responses to drought for plants (Chaves et al., 2003), therefore it is not strange that all populations showed a very pronounced response. Despite the fact that some of the responses described in this study support the theory that selfing could be associated with a dehydration avoidance strategy, in practice, plants combine a range of responses to environmental water limitations (Chaves et al., 2003). Based on our results, water use efficiency and stomatal conductance show no pattern relating selfing rate with dehydration avoidance traits. In contrast (Dudley et al., 2015), observed that in plants of the genus *Clarkia*, a reduction in precipitation caused an increase in drought stress that was reflected by a decline in gas exchange rates, which is consistent with our results. They also found support for the “drought escape” hypothesis, since the study species has a selfing mating system (Dudley et al., 2015).

SLA showed a positive linear relationship with selfing; however, from the triple interaction there can be seen that this positive relationship occurs for *S. grahamii* and not for *S. hookeri* (**Figure 5**). It is widely recognized that a reduction in SLA in plants is aimed at reducing evaporative water losses and enhancing iWUE (Dudley, 1996; Lázaro-Nogal et al., 2015). A decrease in SLA may occur in response to drought in herbaceous leaves as a result of an increased investment in structural tissues allowing increased resistance to unfavorable environmental conditions (Maroco et al., 2000; Chaves et al., 2003). This trait has been widely used in experiments with different water availability regimes both for the comparison of populations within a species, and comparisons between two or more species since it plays an important role in linking plant carbon and water cycles (Marron et al., 2003; Xu and Zhou, 2008; Grassein et al., 2010; Lázaro-Nogal et al., 2015; Wellstein et al., 2017; Dickman et al., 2019).

Therefore, our results suggest that *S. hookeri*, which had lower SLA than *S. grahamii,* appears to show greater dehydration tolerance than *S. grahamii*. However, contrary to our expectations, SLA was lower in the wet treatment than in the dry treatment (**Figure 5**); it has been proposed that a low SLA could be the consequence of high irradiation. The PAR at the greenhouse during these experiment was between 800 and 1,000 μmol m^‑2^ s^‑1^, however, previous studies have shown that PAR can be as high as 1800 μmol m^‑2^ s^‑1^ in the natural habitat of these populations (Casanova-Katny et al., 2006). Another explanation of this result is that, since leaves were measured at the end of the experiment, old plants were not as able to display water saving strategies.

Leaf water content results show a similar pattern, with *S. grahamii* having higher leaf water content than *S. hookeri*, a higher leaf water content has also been previously associated with a dehydration avoidance strategy (Suplick-Ploense and Qian, 2005).

The response of the La Parva population (**Figure 1**) suggests that high levels of selfing may be related to a low survival, regardless of the degree of drought exposure. Although a number of factors might explain this relationship, it may be that higher inbreeding depression leads to increased mortality in highly inbred populations. It is well known that survival is one of the main components of fitness; thus, low survival rates for high selfing could be a sign of a more general pattern of low survival associated with inbreeding in these populations. Although we did not explicitly test for inbreeding depression (Jordan and Otto, 2012), found that high inbreeding depression could be associated to mixed mating species such as *S. grahamii*. This represents a promising area for future research that may elucidate mechanisms underlying distinct patterns observed between the two species. For example, *S. grahamii* populations had an overall lower survival rate in the drought treatment than *S. hookeri* populations. Since final survival was measured after the whole growth season had ended, this pattern could be associated to a dehydration avoidance strategy also called dehydration postponement, this strategy is associated to an enhancement of growth through water acquisition based on “fast” plant traits, which confers drought resistance but not drought survival, therefore a strategy such as this one would prevent them to survive long in drought conditions when forcedly exposed to them. This hypothesis is also supported by the fact that *S. grahamii* had greater leaf area ($\bar{\text{x}}$ = 4.47, se = 0.18) than *S. hookeri* ($\bar{\text{x}}$ = 4.07, se = 0.36), however, within the species, leaf was lower in the highest selfing populations, indicating that this is not the only factor involved in the low survival rates of these populations (**Table S6**,**Figure S2**).

The time limitation hypothesis (Aarssen, 2000; Snell and Aarssen, 2005; Elle et al., 2010; Emms et al., 2018) describes a mechanism by which selfing could be associated with a dehydration escape strategy through the development of smaller flowers, due to a faster life cycle and shorter period of flower development (Elle et al., 2010; Mazer et al., 2010). We hypothesized that his mechanism would only be found in *S. grahamii*, since it has an evolved delayed selfing strategy that has been previously associated with smaller flowers that facilitate selfing at the end of the season (Pérez et al., 2009; Pérez, 2011). It is important to note that we were not able to measure phenology in this study. These are montane species that need certain environmental conditions to flower. In order for them to flower in greenhouse conditions, flowering would've had to be induced. Given the nature and goals our study we decided not to induce flowering since it could've affected our conclusions. However, our findings support this hypothesis through the measurement of “fast” development traits associated with dehydration avoidance. We found that *S. grahamii* had higher growth and photosynthetic rates associated with faster life cycles. *S. grahamii* also showed less survival to the dry treatment and higher SLA and leaf water content, both an indication a low dehydration tolerance, supporting the idea of a physiological trade-off between a dehydration avoidance and dehydration tolerance. This could imply that highly selfing species could be facing a future where they will be more vulnerable if we are to expect even shorter growth seasons that would inevitably expose them to drought during their development. Alternatively, previous studies addressing the evolution of delayed selfing suggest that a strategy that considers both outcrossing and delayed selfing as means of reproduction could report the highest fitness benefits in populations living in unpredictable environments (Herlihy and Eckert, 2002; Jordan and Otto, 2012). Therefore, in the short term it is possible that selection could favor delayed selfing in these new variable and unpredictable environments.

It has been suggested that the frequency of different plant mating systems varies among floras globally (Lloyd, 1980), but the extent to which such patterns exist and are driven by the biogeography of plant–pollinator interactions *versus* other factors remains unknown (Moeller et al., 2017). Our findings offer some evidence to support the importance of abiotic factors such as drought on the possible variation of selfing rates in natural populations and the effect that this mating system could have in their ability to face new environmental conditions such as those imposed by climate change.

## Data Availability Statement

The datasets generated for this study are available on request to the corresponding author.

## Author Contributions

NR, FB and FP originally formulated the idea. NR, FP and FV conceived and designed the experiments. NR and CB performed the experiments. NR analyzed the data. NR and FP wrote the manuscript.

## Funding

This work was funded by CONICYT PIA/BASAL FB0002, CONICYT; FONDECYT N 1171369; doctoral scholarship N° 21110426 granted to NR and FPU scholarship N° AP2010-5600 granted to CB.

## Conflict of Interest

The authors declare that the research was conducted in the absence of any commercial or financial relationships that could be construed as a potential conflict of interest.
