# Supplementary material for: Selfing and Drought-Stress Strategies Under Water Deficit for Two Herbaceous Species in the South American Andes
Source: Front Plant Sci. 2019 Dec 17;10:1595. doi: 10.3389/fpls.2019.01595 (PMC6927913; doi:10.3389/fpls.2019.01595)
Supplement: Supplementary file 1 [file DataSheet_1.docx]

Supplementary Material

**Table S1:** Summer precipitation average and standard deviation between 1930 and 2016, obtained from meteorological stations data and analyzed by CR2 (Centro del Clima y la Resiliencia. DGF. Universidad de Chile)

| Population | Daily Average of summer precipitation | Daily Standard Deviation of summer precipitation |
| --- | --- | --- |
| La Parva | 0,4 mm | 2,4 mm |
| Valle Nevado | 0,4 mm | 2,4 mm |
| Lagunillas | 0,4 mm | 2,5 mm |
| Laguna los Cristales | 0,5 mm | 3,2 mm |
| Teno | 0,9 mm | 5,6 mm |

**Table S2:** Results from the ANOVA analysis for daily growth rate. F-values are shown, and degrees of freedom are below every factor in parenthesis.

|  | Treatment  (d.f = 1) | Selfing  (d.f = 1) | Species  (d.f = 1) | Selfing x Species  (d.f =1) | Error d.f |
| --- | --- | --- | --- | --- | --- |
| Daily Growth rate | 0.005 | 0.264 | 10.7** | 6.66* | 821 |

Significance levels: P > 0.05 (ns = not significant); P < 0.05 = * ; P< 0.01 = **; P < 0.001 = ***

**Table S3:** Results from the ANOVA analysis for stomatal conductance. F-values are shown, and degrees of freedom are below every factor in parenthesis.

|  | Treatment  (d.f = 1) | Selfing  (d.f = 1) | Species  (d.f = 1) | Error d.f |
| --- | --- | --- | --- | --- |
| Stomatal conductance | 335.39*** | 0.54 | 1.04 | 172 |

Significance levels: P > 0.05 (ns = not significant); P < 0.05 = * ; P< 0.01 = **; P < 0.001 = ***

**Table S4:** Results from the ANOVA analysis for water use efficiency (WUE). F-values are shown, and degrees of freedom are below every factor in parenthesis.

|  | Treatment  (d.f = 1) | Selfing  (d.f = 1) | Species  (d.f = 1) | Error d.f |
| --- | --- | --- | --- | --- |
| Water use efficiency | 221.4*** | 1.81 | 1.29 | 171 |

Significance levels: P > 0.05 (ns = not significant); P < 0.05 = * ; P< 0.01 = **; P < 0.001 = ***

**Table S5:** Results from the ANOVA analysis for leaf water content. F-values are shown, and degrees of freedom are below every factor in parenthesis.

|  | Treatment  (d.f = 1) | Selfing  (d.f = 1) | Species  (d.f = 1) | Selfing x Species  (d.f =1) | Error d.f |
| --- | --- | --- | --- | --- | --- |
| Leaf water content | 1.68 | 18.98^***^ | 21.06^***^ | 25.95^***^ | 703 |

Significance levels: P > 0.05 (ns = not significant); P < 0.05 = * ; P< 0.01 = **; P < 0.001 = ***

**Table S6:** Results from the ANOVA analysis for mean leaf area. F-values are shown, and degrees of freedom are below every factor in parenthesis.

|  | Treatment  (d.f = 1) | Selfing  (d.f = 1) | Species  (d.f = 1) | Selfing x Species  (d.f =1) | Error d.f |
| --- | --- | --- | --- | --- | --- |
| Mean leaf area | 11.37^***^ | 0.02 | 17.91^***^ | 8.81^**^ | 750 |

Significance levels: P > 0.05 (ns = not significant); P < 0.05 = * ; P< 0.01 = **; P < 0.001 = ***

**Figure Legends:**

**Figure S1**: Relationship between Leaf Water Content (g) and selfing rate for *Schizanthus hookeri* (left) and *Schizanthus grahamii* (right). Red dots are measurements for each selfing rate at the dry treatment and green dots are measurements for each selfing rate at the wet treatment, lines represent linear relationships (red: dry treatment, green: wet treatment), and gray shaded areas represent standard error. S.H., Schizanthus hookeri; S.G., Schizanthus grahamii; D, dry treatment; W, wet treatment.

**Figure S2:** Relationship between Leaf Area (cm) and selfing rate for *Schizanthus hookeri* (left) and *Schizanthus grahamii* (right). Red dots are measurements for each selfing rate at the dry treatment and green dots are measurements for each selfing rate at the wet treatment, lines represent linear relationships (red: dry treatment, green: wet treatment), and gray shaded areas represent standard error. S.H., Schizanthus hookeri; S.G., Schizanthus grahamii; D, dry treatment; W, wet treatment.

**Fig S1**

**Fig S2:**
